# Relationship between retinal capillary vessel density of OCT angiography and intraocular pressure in pig

**DOI:** 10.1038/s41598-021-87689-8

**Published:** 2021-04-20

**Authors:** Mihyun Choi, Seong-Woo Kim, Somin Ahn, Thi Que Anh Vu, Cheolmin Yun, Yong Yeon Kim

**Affiliations:** 1 Department of Ophthalmology, Korea University Guro Hospital, 148, Gurodong-ro, Guro-gu, Seoul, 08308 Republic of Korea; 2 Department of Ophthalmology, Hanoi Medical University, Hanoi, Vietnam; 3 Department of Ophthalmology, Korea University Ansan Hospital, 123, Jeokgeum-ro, Danwon-gu, Ansan-si, Gyeonggi-do Republic of Korea

**Keywords:** Anatomy, Medical research

## Abstract

The purpose of this study was to evaluate density change in the retinal capillary plexus during intra ocular pressure (IOP) elevation in vitrectomized pigs’ eyes using optical coherence tomography angiography (OCTA). Eight eyes of eight micro pigs received vitrectomy and the IOP was controlled from 15 mmHg (baseline) to 105 mmHg in 15 mmHg increments using a vented-gas forced-infusion system, and then decreased back to normal IOP (recovery state). The spectral-domain OCTA device was set to scan an area of 8.8 × 4.4 mm (30° × 15°) above the optic nerve head for each IOP. The relative vessel density (rVAD) compared to baseline was determined for the total retinal blood flow (RBF) which included major retinal artery and venous vessels, radial peripapillary capillaries (RPCs), superficial (SVP), intermediate (IVP), and deep vascular plexus (DVP). The mean rVAD was 0.890 in RBF, 0.826 in RPCs, 0.817 in SVP, 0.819 in IVP, and 0.794 in DVP at 30 mmHg. While the rVAD of RBF and RPCs decreased to 0.504 and 0.541 at 45 mmHg, the SVP, IVP, and DVP decreased to 0.433, 0.359, and 0.345, respectively. When IOP was normalized, the rVAD was recovered in all layers and the VAD of RBF, IVP, and DVP were higher than baseline (P = 0.040, 0.019, and 0.019, respectively). Retinal capillary density deterioration in each layer was found from 30 mmHg using an OCTA system which showed excellent depth-resolved segmentation of retinal capillary layers even at higher IOPs. Reduction in VAD showed full recovery after IOP normalization.

## Introduction

Blood flow that supplies oxygen and nutrients is essential for proper retinal function. The holangiotic microcirculation of the mammalian retina consists primarily of parallel intra-retinal layers of microvessels and peri-papillary capillaries around the disc^1–3^. Confocal microscopy and three dimensional reconstruction of the retina in human and animal models has provided significant information about the serial organization of the retinal capillary networks^1,4^. Human retinal capillaries that comprise the superficial vascular complex consist of the superficial vascular plexus (SVP) and retinal peripapillary capillaries (RPCs) in nerve fiber and ganglion cell layers and intermediate (IVP) and deep vascular plexuses (DVP) in the inner plexiform and outer plexiform layers, respectively^5^. These plexuses represent terminal anastomotic capillary networks supplied by vertically oriented interconnecting arterioles and venules of the SVP^6^.

Pig retinas have a similar vascular structure to human retinas, but instead of the central retinal artery, several cilioretinal arteries emerge from the optic nerve head and divide into four or five major branches (major cilioretinal vessels)^7,8^. The retinal blood flow from major arteries runs exclusively into the superficial vascular plexus (SVP), which divides into radial peripapillary capillaries (RPCs) on one side (inner) and the intermediate (IVP) and deep vascular plexuses (DVP) on the other side (outer)^1^. Because each retinal capillary plexus (SVP, IVP, and DVP) except the RPCs has a hammock-like structure and does not make direct connections to the others, the flow is serially organized to flow from the SVP to the IVP and DVP. Radial peripapillary capillaries (RPCs) run parallel to the nerve fiber layer (NFL) around the disc and drain to the IVP or DVP^3^. The similarity of these microstructures makes the pig a good experimental model for hemodynamic analysis of intra-retinal microcirculation in humans.

Elevated intraocular pressure (IOP) is the most important factor that contributes to mechanical and ischemic damage to the optic nerve head and retinal capillary perfusion. During ophthalmic surgery such as vitrectomy, an increase in intraocular pressure beyond the physiological range can be induced (80–100 mmHg)^9^, and this can lead to retinal capillary perfusion damage. However, there is a shortage of experimental analyses of intra-retinal capillary perfusion according to IOP change in large eyes. Numerous non-invasive techniques previously used to evaluate ocular circulation include measuring the retinal vessel caliber^10^ or time of circulation in fluorescein angiograms^11–13^. Laser Doppler velocimetry, Laser Doppler flowmetry, and laser speckle flowgraphy were also used as standard methods to evaluate blood flow in the retina; however, they are limited by their inability to distinguish the different retinal capillary layers^14^. Recently, the innovative development of optical coherence tomography angiography (OCTA) has allowed rapid progress in visualization of the vascular networks in living subjects by analyzing the dynamic signals generated by moving red blood cells (RBC) in perfused blood vessels^15–17^.

In this study, we observed the changes in capillary density in each capillary plexus according to IOP change through OCTA in a pig model, which is a large animal who has vascular similarity to humans.

## Results

The mean axial length of eyeball was 19.53 ± 0.58 mm (18.80–20.46) and baseline IOP before vitrectomy was 9.70 ± 1.87 mmHg (7.26–12.76). Mean systolic blood pressure was 83.85 ± 9.66 mmHg (73–103) and mean diastolic blood pressure was 40.81 ± 8.4 mmHg (60–35). After increasing the IOP, the time required for each OCTA imaging measurement was 72.3 ± 18.96 s (40–120). The mean IOP in the recovery state was 13.89 ± 4.34 mmHg (6.4–21.1).

### In vivo OCTA imaging of the pig eye

**Figure 1:**
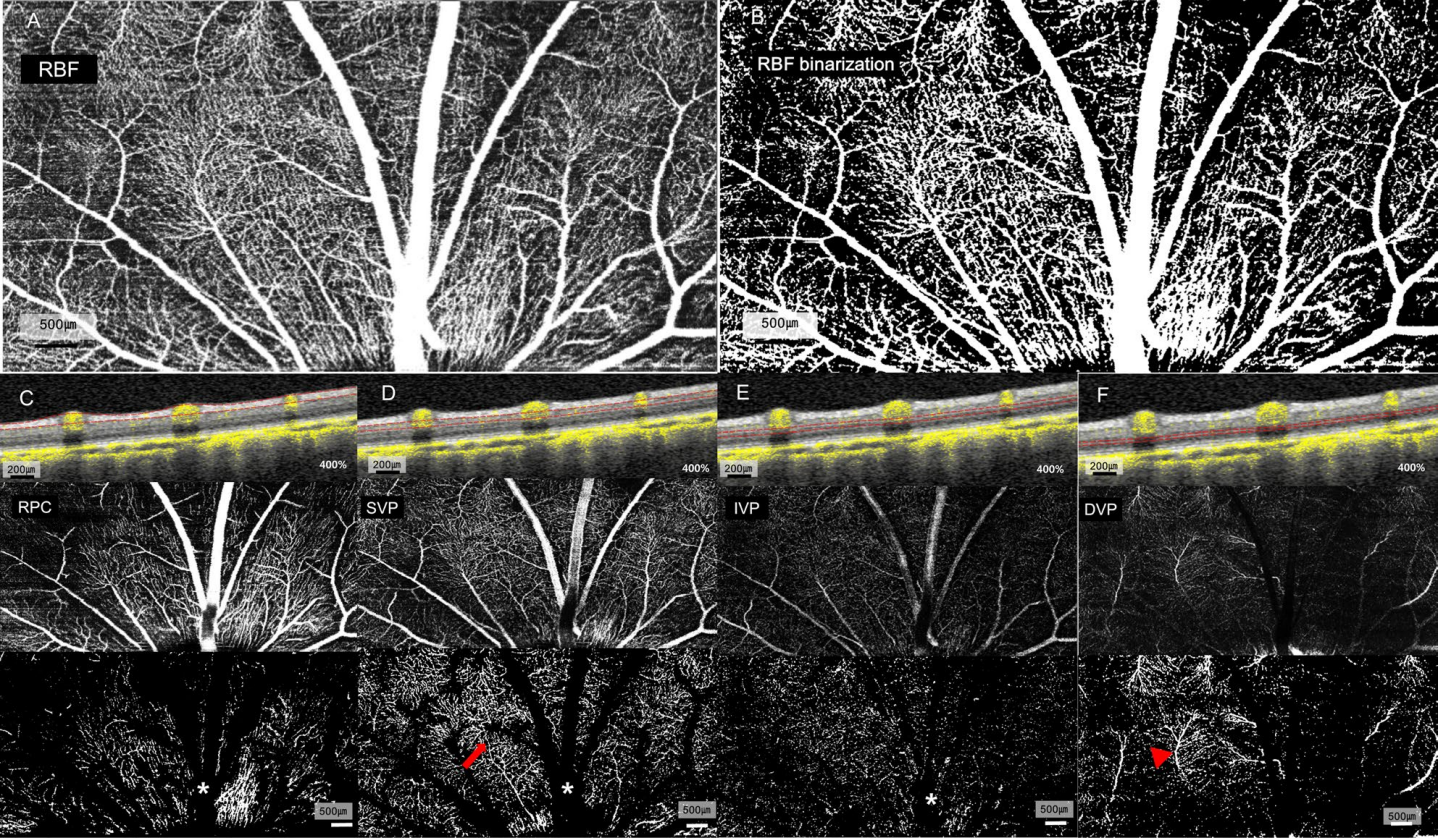
Segmentation of the retinal vascular layer. (**A**) Total retinal blood flow layer from the internal limiting membrane to the outer plexiform layer. (**B**) Binarization image of total retinal blood flow. (**C**) Radial peripapillary capillaries from the inner nuclear layer to the retinal nerve fiber layer showed long capillaries running parallel to the nerve fiber layer. (**D**) Superficial vascular plexus from ganglion cell layer to the inner margin of the inner plexiform layer showed arborescent patterns with central arterioles (red arrow). (**E**) Intermediate vascular plexus from the inner plexiform layer to the inner margin of the inner nuclear layer had no prominent arterioles or venules. (**F**) Deep vascular plexus from the inner nuclear layer to the outer plexiform layer showed prominent central venules (red arrowhead) and arborescent capillaries. (**C**–**F**) (upper) In fusion images of structural OCT section images and the corresponding blood flow information (structural OCT + OCTA) (magnification × 4), direct visual confirmation of structural and flow information (yellowish dot overlay) in each vascular plexus was established by the user. (middle) *En-face* OCTA images and (lower) binarized images of *en-face* OCTA images. Major cilioretinal vessels were manually removed in binarized images of RPC, SVP, IVP (white asterisk in **C**–**E**).

**Figure 2:**
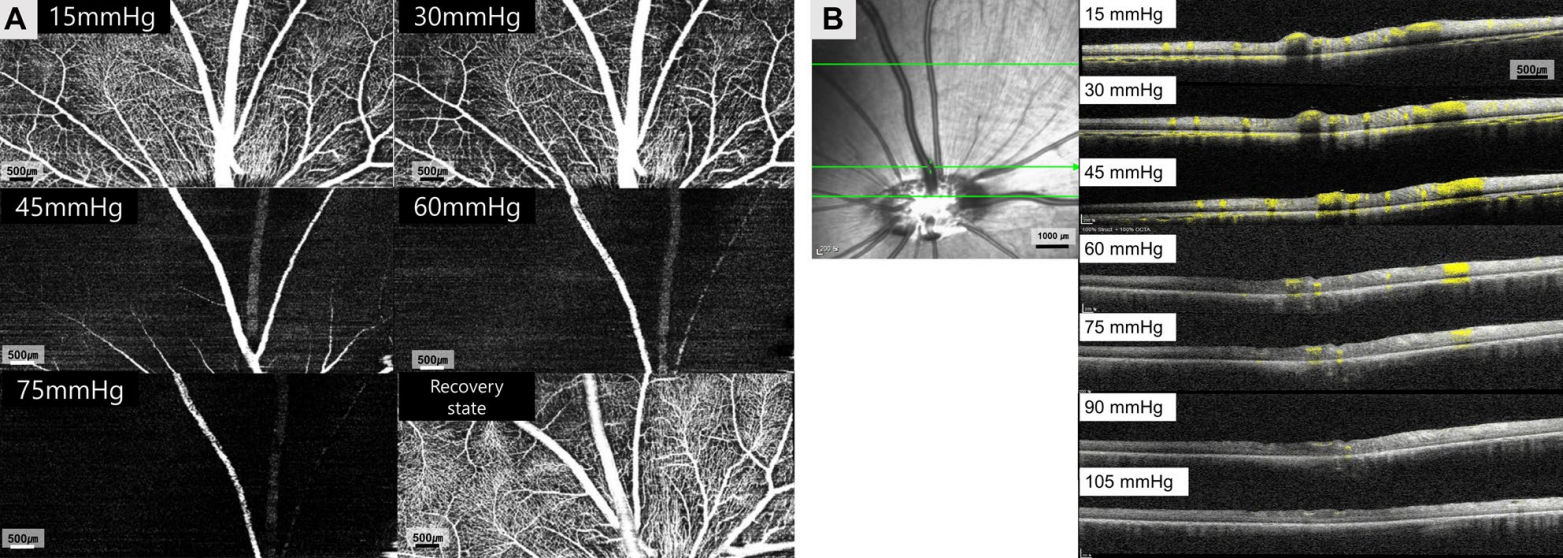
(**A**) Qualitative change in total retinal blood flow according to changes in IOP from 15 to 75 mmHg, and the recovery state. The fine end of capillaries which made inter-capillary connections seemed to be more diminished than capillaries connected to arterioles and venules at 30 mmHg of IOP. At 45 mmHg, a dramatic decrease in capillary flow was observed. Only major cilioretinal vessels and peripapillary vessels showed flow signal above 60 mmHg. In the recovery state, all of the major vessels and capillary flows seemed to recover their signal intensity. (**B**) Structural OCT + OCTA B-scan cross section image shows flow signal (yellowish dot) on structural OCT. When the intraocular pressure rises to 45 mmHg, the lumen of the major retinal vessels started to collapse and the vascular lumen flattened; capillary flow decreases as pressure rise. However, the flow signal still appears to be present in the cilioretinal arteries until an IOP of 75 mmHg. In the choroidal circulation, as shown in the *en-face* OCT, the flow reduction was clearly seen at 45 mmHg, and almost no flow signal was observed above 60 mmHg.

**Figure 3:**
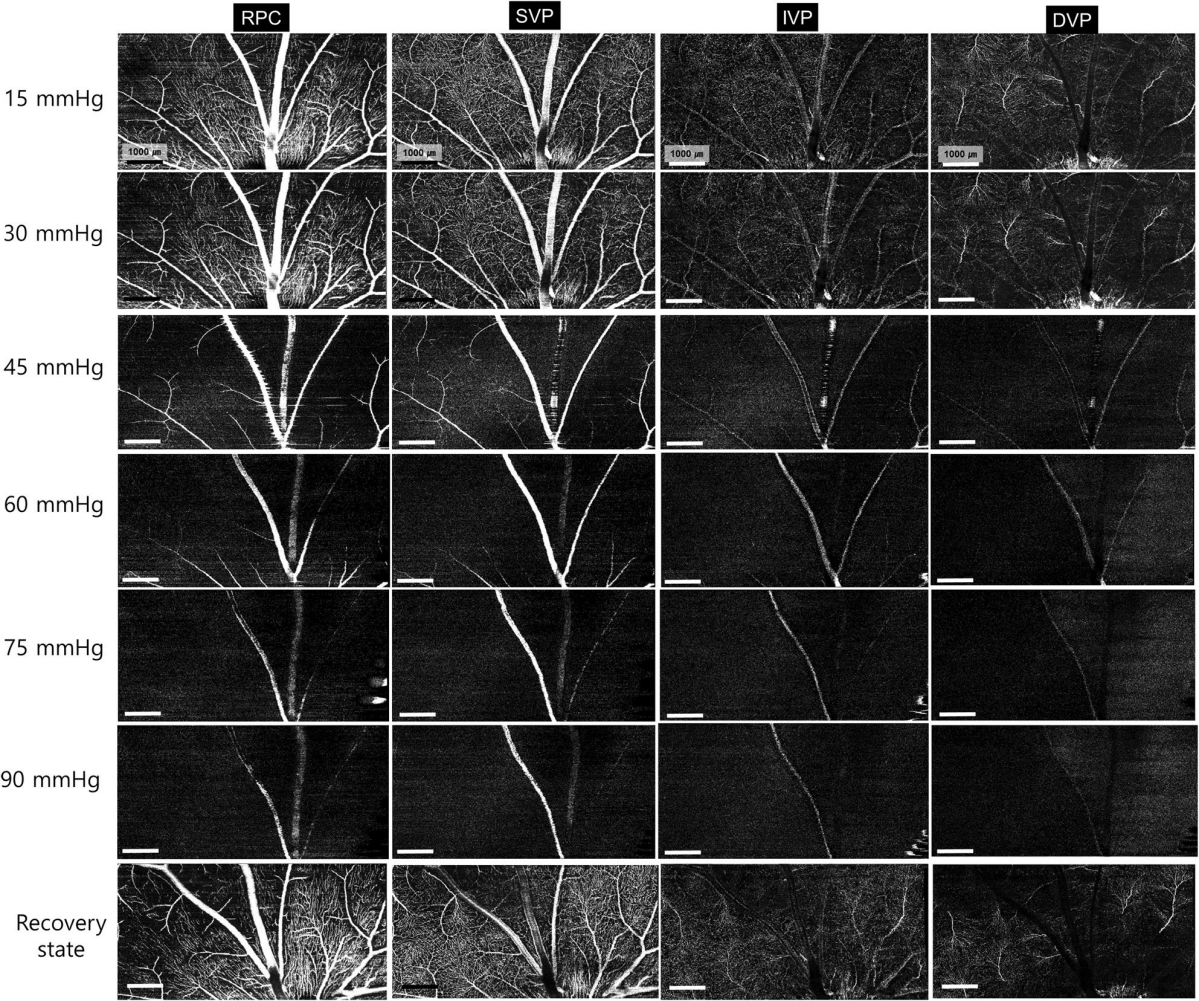
Changes in each retinal and choroidal vascular layer according to changes in IOP from 15 to 90 mmHg, and the recovery state. Retinal capillary attenuation was found at 30 mmHg of IOP. At 45 mmHg, arterioles and venules were observed, but capillary perfusion significantly diminished. In the recovery state, all the retinal capillary layers seemed to have similar signal intensity to baseline. All scale bars indicate 1000 μm.

OCTA *en-face* images demonstrated the resolution necessary to enable real-time analysis of each capillary layer in vivo (Fig. 1). Qualitative changes in total retinal blood flow are shown in Fig. 2A. In the structural OCT + OCTA B-scan cross section image (Fig. 2B) according to IOP, the major retinal vessel lumen was collapsed by IOP above 45 mmHg. The OCTA image of each retinal capillary layer are presented in Fig. 3.

### Quantification of vessel density in OCTA *en-face* images

**Figure 4:**
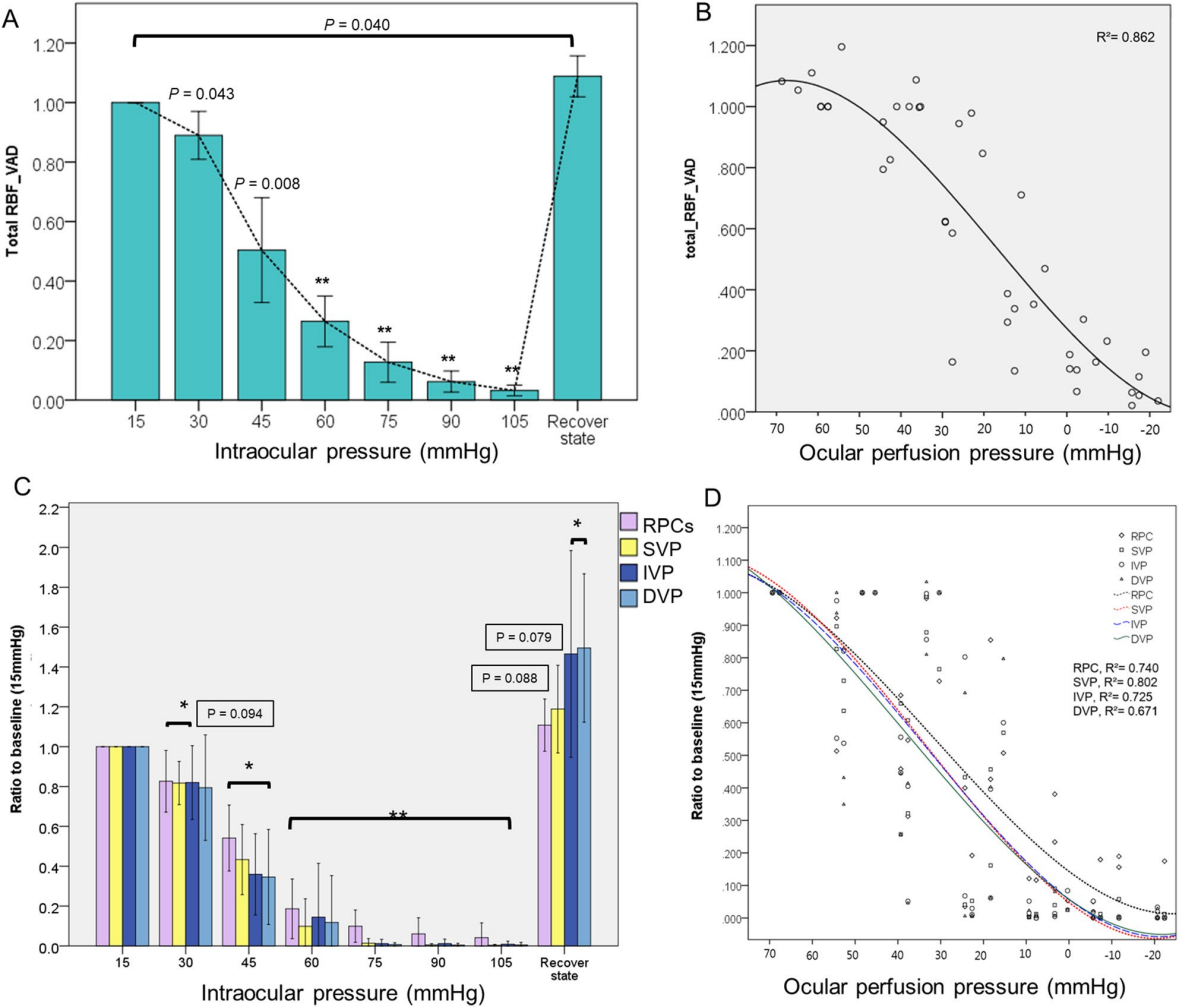
Changes of retinal and choroidal blood flow according to intraocular pressure. (**A**) The relative vessel area density (rVAD) of total retinal blood flow according to intraocular pressure. (**B**) The plotted rVAD of total retinal blood flow according to ocular perfusion pressure. (**C**) The relative vessel area of each retinal capillary layer according to intraocular pressure (*RPCs* radial peripapillary capillaries, *SVP* superficial vascular plexus, *IVP* intermediate vascular plexus, *DVP* deep vascular plexus). (**D**) The plotted rVAD of each retinal capillary layer according to ocular perfusion pressure. IOP levels significantly different from the baseline are indicated by * for P < 0.05 and ** for P < 0.01 in (**A**, **C**). The values of the recovery state is not included in (**B**,** D**).

The ICC of two examiner for perfusion quantification was 0.951 in total retinal blood flow (RBF), 0.922 in RPCs, 0.903 in SVP, 0.953 in IVP and 0.958 in DVP (all P < 0.001). There were no differences in VAD for each capillary layer between OCTA obtained prior to vitrectomy and baseline (Supplementary Table 1). The mean value of relative VAD (rVAD, compared to 15 mmHg baseline) for RPCs, SVP, IVP, and DVP at each IOP level in eight pig are presented in Table 1 and illustrated in Fig. 4. In RBF, the mean rVAD showed a slight reduction (0.890) at 30 mmHg (*P* = 0.043) (Fig. 4A). RBF still maintained half the vessel area density compared to the baseline even at 45 mmHg, but dropped to 10% of baseline at 75 mmHg. In the recovery state, rVAD increased significantly, showing a higher value than the baseline (*P* = 0.040). We plotted rVAD values for RBF according to ocular perfusion pressure (OPP) and found that the total retinal blood vessel density did not decrease more than 20% until the OPP decreased below 40 mmHg, and a 50% reduction in total retinal blood vessel density was observed when OPP decreased to 20 mmHg (Fig. 4B).

**Table 1:** Relative vessel area density compared to baseline (15 mmHg).

IOP (mmHg)	RBF	RCP	SVP	IVP	DVP
**30**	0.890 (0.077)	0.826 (0.167)	0.817 (0.117)	0.819 (0.200)	0.794 (0.287)
P-value	0.043	0.031	0.003	0.028	0.094
**45**	0.504 (0.191)	0.541 (0.179)	0.433 (0.191)	0.359 (0.220)	0.345 (0.258)
P-value	0.008	0.002	0.001	0.002	0.002
**60**	0.265 (0.092)	0.186 (0.162)	0.098 (0.149)	0.144 (0.291)	0.117 (0.254)
P-value	< 0.001				
**75**	0.127 (0.064)	0.098 (0.077)	0.013 (0.022)	0.011 (0.050)	0.006 (0.008)
P-value	< 0.001				
**90**	0.062 (0.034)	0.060 (0.077)	0.005 (0.005)	0.011 (0.020)	0.003 (0.008)
P-value	< 0.001				
**105**	0.032 (0.017)	0.041 (0.071)	0.003 (0.004)	0.008 (0.014)	0.004 (0.011)
P-value	< 0.001				
**Recovery state**	1.088 (0.065)	1.108 (0.124)	1.190 (0.209)	1.465 (0.494)	1.495 (0.354)
P-value	0.040	0.088	0.079	0.019	0.019

All values are presented in mean (standard deviation).

All P-value; repeated ANOVA test, compared with baseline (15 mmHg).

*RBF* total retinal blood flow, *RPCs* radial peripapillary capillaries, *SVP* superficial vascular plexus, *IVP* intermediate vascular plexus, *DVP* deep vascular plexus, *VAD* vessel area density.

The mean rVAD for retinal capillary layers of RPCs, SVP, IVP, and DVP was 0.826, 0.817, 0.819, and 0.794 at 30 mmHg, respectively, which showed greater reduction than baseline although the change in DVP was not statistically significant (P = 0.094) (Table 1 and Fig. 4C). RPC showed a relatively gradual decrease in rVAD at IOP greater than 45 mmHg. It is noteworthy that the rVAD of IVP and DVP in the recovery state were increased over baseline (all P < 0.05). In the RPC and SVP, VAD increased in the recovery state, but was not statistically different from the baseline (P = 0.088 and P = 0.079). All four capillary layers showed about a 20% reduction in VAD at an OPP of 50 mmHg, and a 50% reduction was observed with an OPP of 30 mmHg, which showed earlier reduction of perfusion than total retinal blood flow which includes major cilioretinal vessels (Fig. 4D).

### Vascular staining and confocal images

**Figure 5:**
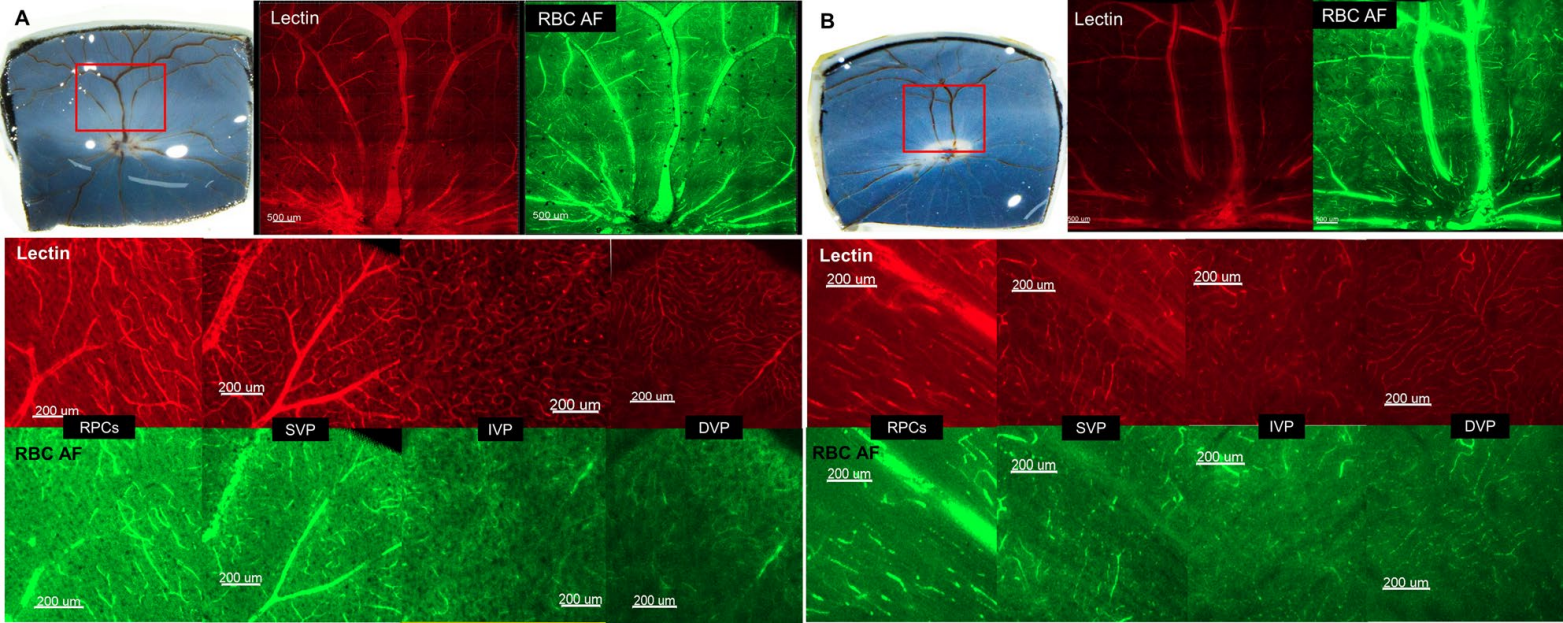
Tissue staining of retinal capillary layers at baseline (**A**) and 90 mmHg of IOP (**B**). The tissue area examined by OCTA was confirmed in gross specimens (upper left) and then lectin staining was performed. The entire tissue was divided into 25 tiles and orthogonal confocal images (× 5 magnification) of the lectin stain (upper middle) and RBC autofluorescence (upper right) were obtained. (second and third rows) *En face* view of the different capillary levels of the pig retina (upper: lectin-stained images, lower: RBC autofluorescence images).

The lectin stains fixed at 15 mmHg and 90 mmHg are shown in Fig. 5A,B. In segmented images of each capillary layer at 90 mmHg, all layers showed a partially-destroyed structure especially in the capillary end and had attenuated, narrowed and obliterated vascular structures compared to an IOP of 15 mmHg. However, images of RBC autofluorescence in Fig. 5 showed no remarkable changes according to IOP.

## Discussion

IOP elevation can occur during various ophthalmic diseases including glaucoma and ophthalmic surgical procedures, leading to optic nerve damage and perfusion reduction of retinal and optic nerve heads^18,19^. Experimental models demonstrated IOP fluctuations during vitrectomy, ranging from 0 to 120 mmHg^9^, and high IOP (80 mmHg) during vitrectomy can lead to damage of retinal ganglion cells in a rabbit model^20^. In the present study, we presented an experimental model for measuring retinal capillary layer vessel density according to IOP rise using OCTA in pig, which have similar serially organized flow within layers of capillary plexus to humans. Previous studies using OCTA, have demonstrated a decrease in perfusion around the optic nerve head of small animals such as rats^21,22^, but no studies have been conducted in large mammalian animal such as pig. We focused on VAD at the capillary level, which excluded cilioretinal arteries and veins. Further, in previous experiments, the pressure transducer cannula was inserted in the anterior chamber^23^. We provided pressure after vitrectomy to directly control IOP to the retina without vitreous and to maintain anterior chamber stability.

Xu et al. obtained OCTA images of the optic nerve head while increasing IOP by 10 mmHg increments in rats and reported that the total retinal blood perfusion decreased to 90% of baseline at 30 mmHg and VAD reached 50% of baseline at 60 mmHg^24^. In our study, we have also shown that mean rVAD of total retinal blood flow was 0.890 at 30 mmHg, similar to a previous study, but VAD for each retinal capillary layer excluding major cilioretinal vessels showed greater deterioration at 30 mmHg than in total retinal blood flow. In particular, at 45 mmHg of IOP, VAD in SVP, IVP, and DVP decreased below 50% compared to baseline, suggesting that the intra-retinal capillary layer was more vulnerable to IOP rise. Of the retinal capillary layers, RPCs showed a relatively gradual decrease in VAD. The RPCs have relatively linear vessels on the retinal surface along ganglion cell axon fascicles, are supplied blood directly from the SVP, and rarely anastomose. These morphological features might attribute to make the RPCs less likely to be affected by IOP than other intra-retinal capillary layers.

Retinal vessels have a mechanism to control local vascular diameters that is maintained by muscular tonus of the arterioles and contraction of the pericytes^25,26^. Studies in human subjects suggest that these auto-regulatory mechanisms break down at a threshold IOP above 30 mmHg^27,28^ which parallels our observations of total RBF relative preservation at 30 mmHg. According to Nagel et al., when the IOP increases, retinal arterial dilation occurs within seconds and the venous diameter, given its relatively weaker wall, decreases, thereby increasing venous outflow resistance^29^. While keeping the IOP high, the venous diameter gradually increased to the baseline level (before IOP elevation) through smooth muscle cell relaxation in the vessel wall. When the IOP normalized, the dilated arterial vessel returned to baseline shortly afterwards, but the veins showed sudden dilation due to relaxed smooth muscle cells for several minutes. In our experiments, it was speculated that the increases in VAD of RBF and each retinal capillary plexus layer were caused by venous dilation in the recovery state. To define the mechanism of this increase in VAD, further experiments with adjustment of exposure time to increase intraocular pressure is necessary.

There are some limitations to this study, including the fact that OCTA images are affected by projection artifact due to by major cilioretinal vessels. The blood pressures were not obtained through direct arterial catheterization, which is a gold standard, but measured indirectly by tail cuff which showed good correlation with direct measurement^30^. The overall blood pressures during the experiment were measured to be about 10 mmHg lower than previously reported values^31^. This may be due to the measurement method, or may be caused by placing the pig in the lateral decubitus position during the vitrectomy procedure. We also know that vessel density measurements of binarized OCTA images which were selected for segmentation of each retinal capillary plexus do not fully reflect blood flow. In addition, efforts were made to obtain segmentation of the capillary plexuses by manually removing the large vessel signal and applying the manufacturer’s projection artifact rejection algorithm; projection artifacts are still a problem in quantitative measurement of perfused capillaries.

In summary, we demonstrated changes of retinal vessel density according to IOP rise and recovery using an OCTA system and found more vulnerable change of VAD to IOP elevation in retinal capillary layers than in major cilioretinal vessels. OCTA showed excellent depth-resolved segmentation of capillary layers of the retina even at higher IOPs in a non-invasive and reproducible way. We believe this achievement of OCTA can provide additional methods for capillary perfusion evaluation and is highly useful in ophthalmic diseases including glaucoma.

## Methods

### Animal preparation

This study was carried out in compliance with the ARRIVE guidelines and performed in accordance with the National Institutes of Health for the Care and Use of Laboratory Animals (NIH Publications 104 No. 8023, revised 1978) and approved by the Institutional Animal Care and Use Committee of Korea University College of Medicine. The subjects of the experiment were eight female micro-pigs (APURES Co., LTD., Pyeongtaek, Korea) bred in a specific pathogen-free area, at approximately 47 ± 10.71 (minimum–maximum, 37–59) weeks of age with a mean weight 28.47 ± 3.88 (23.0–33.2) kg. The pig underwent general anesthesia by an intravenous injection of alfaxalone (1 mg/kg; Alfaxan, Vetoquinol, West Sussex, UK) into the marginal auricular vein following premedication including subcutaneous injection of atropine (0.05 mg/kg) and intramuscular injection of xylazine (0.5 mg/kg; Rompun, Bayer Corp., Shawnee Mission, KA, USA) and azaperone (2 mg/kg). After sufficient sedation, the pigs were placed in the supine position and intubated with an orotracheal tube (6.0–6.5 French size). Anesthesia was maintained with isoflurane (1.5–2% Isoflurane, ISOTROY 250, Troikaa Pharmaceuticals Ltd., Gujarat, India) and proper hydration was retained with normal saline (2–3 mL/kg/h). Mean arterial blood pressure (MAP) was monitored non-invasively by tail cuff (Neonate #2, NT Plus, MEK, Korea) and respiratory rate, pulse, and blood oxygen saturation were monitored. After general anesthesia, the axial length and baseline IOP were measured five times using A-scan ultrasound biometry (SW-1000, Suoer, China) and a rebound tonometer (Icare ic200, Icare Finland Oy, Helsinki, Finland)^32^, and the eye was irrigated with 5% povidone-iodine and draped for the experiment.

### Vitrectomy and control of IOP

A three-port, 23-gauge vitrectomy (Associate; DORC, Zuidland, Netherlands) was performed in eight right eyes of eight pigs to apply external pressure directly to the retina. Three ports were prepared by inserting trocar cannulas into the sclera 3 mm from the limbus at the ventromedial, ventrolateral, and dorsolateral sides. An infusion line which continuously supplied balanced salt solution (BSS; Alcon, Fort Worth, TX) was connected at the dorsolateral port. Total vitrectomy with posterior vitreous detachment was performed with an indirect lens (Oculus BIOM ready, Oculus Surgical, Inc., FL, USA) in all subjects. To obtain clear images without media opacity, an anterior lens capsule-saving lensectomy was performed. After the peripheral vitrectomy, all ports except the dorsolateral port (connected to the infusion) were removed and the perforated sclera sites were sutured.

**Figure 6:**
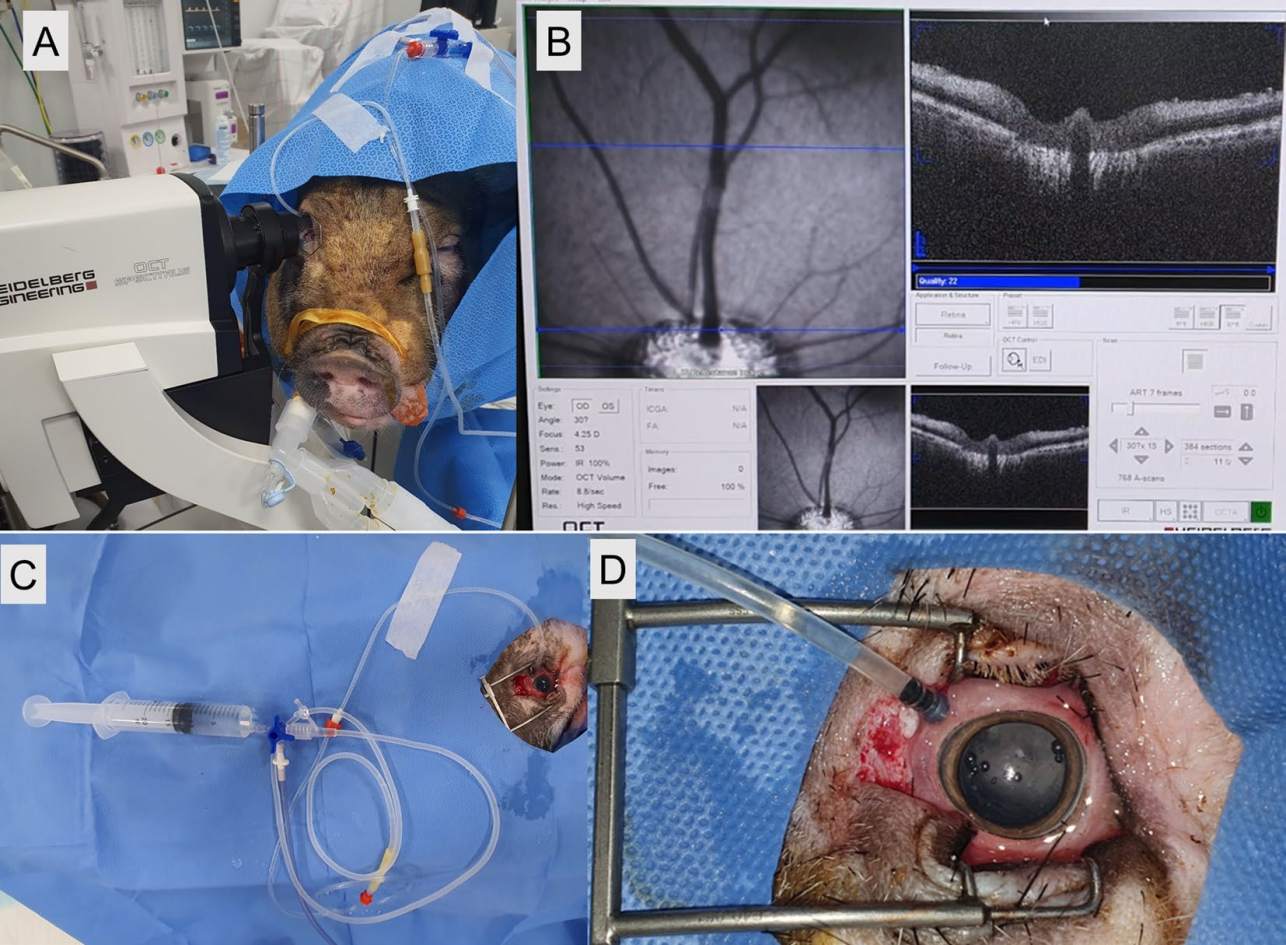
The experimental procedures. (**A**) The vitrectomy was performed in the decubitus position and pig was positioned to lay straight for OCTA imaging. A ventrolateral infusion was maintained and connected to the vented-gas forced-infusion system. (**B**) The position of the eye during OCTA image capture. The optic nerve head was maintained in the lower center of the fundus region and visualized with the scanning-laser ophthalmoscopy module. (**C**, **D**) Tissue fixing procedure. The zinc formalin fully filled the eye and continuous pressure was supplied by the VGFI system.

A three-way connector was added to the BSS infusion line; the vented-gas forced-infusion (VGFI; Associate; DORC, Zuidland, Netherlands) could directly apply the designated pressure into the vitreous cavity through this connector in response to input by the operator. The three-way connector and infusion line were more than 50 cm from the scleral eyeball port and this line was filled with BSS to avoid air injection into vitreous cavity, which may interfere with retinal image acquisition (Fig. 6A). By converting the three-way connector to VGFI, we created a system directly capable of regulating IOP. This system has previously been shown to reliably produce an IOP equivalent to that indicated by the VGFI system^33^.

### OCTA image acquisition

OCTA was obtained via a spectral-domain OCT device (Spectralis OCT2, Version 1.10.4.0; Heidelberg Engineering, Heidelberg, Germany) set to scan an area of 8.8 × 4.4 mm (30° × 15°). The eye was maintained with the optic nerve head in the lower center of the fundus region, as visualized with the scanning-laser ophthalmoscopy module of the OCT device, and cilioretinal arteries were located in the center of the image (Fig. 6B). A rigid contact lens (base curve: 9.6) of zero power was applied on the cornea to avoid corneal dryness and haziness during image acquisition. OCTA images were obtained from all pig prior to vitrectomy (control). After vitrectomy as described above, IOP was elevated from 15 to 105 mmHg in 15 mmHg increments, and OCTA imaging was obtained at each IOP after 2 min. After taking OCTA images at 105 mmHg of IOP, the infusion line of the dorsolateral port was removed (reserve dorsolateral port) and the IOP was measured approximately 20 min later. This was defined as “recovery state” and OCTA imaging was re-obtained at this point.

### Tissue fixing in living pig

After the OCTA was obtained up to the “recovery state”, the vitreous cavity was tamponaded again with zinc formalin (Zinc Formalin Fixative, pH 6.25, Polyscicences, Inc., PA, USA) (Fig. 6C,D) after fluid-air exchange. Then, we set the IOP differently for each eye in seven subjects (at 15, 30, 45, 60, 75, 90, and 105 mmHg) with the VGFI system to fix the intraocular tissue, under the designated IOP. The IOP was checked every 10 min using a rebound tonometer to confirm that constant IOP was applied. After 1 h of tamponade, the dorsolateral port was removed and scleral suture was performed. Immediately after euthanasia, the subject eyes were enucleated and immersion-fixed in zinc formalin.

### Vascular staining

After removing the corneo-scleral rim from the enucleated eyeball, a 15 mm (horizontal) × 12 mm (vertical) incision was made in the ocular tissue above the disc to include the location where the OCTA images were taken. Vascular staining was performed without separate isolation of the retinal-scleral layer. Retinas were then washed with PBS for ten intervals of 10 min and placed in lectin stain (Licopersicon Esculentum Lectin FSD Fluor 647) at a ratio of 1:100 with PBS for 3 days, followed by rinsing with PBS for three intervals of 30 min. Retinas were incubated in Binaree Mounting solution (SHMS-060) for 1 day and then mounted on chamber slides.

Stained retinas were imaged using a laser confocal microscope (ZEISS LSM 800) equipped with Plan-Apochromat 10X/0.45NA. Images were sampled at a resolution of 512 × 512 pixels with a *z*-step size of 3 μm. The retinal blood vessels containing auto-fluorescent red blood cells were imaged by confocal microscopy (excitation ~ 470 nm). Images were generated by using the Zeiss tiling function, which creates a large-volume image by stitching together multiple highly resolved and magnified individual images. *3D* visualization and image processing were processed using Imaris software (Oxford instruments).

### OCTA image processing and analysis

The automated segmentation of the en-face OCTA images (HEYEX; Heidelberg Engineering, Heidelberg, Germany) were performed. In order to measure the total retinal blood flow including major cilioretinal vessels and all capillary layers in previous studies, the RBF layer was included from the internal limiting membrane (ILM) to the outer plexiform layer (OPL) (Fig. 1A,B)^21,24^. Each retinal capillary layer was defined as below; RPCs from the ILM to the retinal nerve fiber layer (NFL) (Fig. 1C); SVP from the ganglion cell layer (GCL) to the inner margin of the inner plexiform layer (IPL) (Fig. 1D); IVP from the IPL to the inner margin of the inner nuclear layer (INL) (Fig. 1E); and DVP from the INL to the OPL (Fig. 1F)^5^. In B-scan images co-registered with structural OCT scans, the blood flow signal of OCTA and auto-segmentation line were confirmed by a retinal specialist (M. C.). The auto-segmented line for en-face images was manually adjusted as needed. To quantify retinal microvascular perfusion at each IOP, vessel area density (VAD) was adopted^34^. To calculate VAD, *en-face* images were binarized using ImageJ software (National Institutes of Health, Bethesda, MD, USA). In the RPC, SVP, and IVP images, there were major cilioretinal vessels originating from the disc, which can lead to and overestimation of the capillary vessel density. To minimize the effects of these large cilioretinal vessels, the signal in major cilioretinal vessels (> 30 μm of diameter) was excluded using the wand tool of image J software on RPC, SVP and IVP images after binarization (bottom images of Fig. 1C–F).

Due to the refractive index of the lens, the presence of lens tissue might induce differences in vessel analysis of OCTA. Therefore, an IOP of 15 mmHg was set as the baseline after vitrectomy and lensectomy. We compared the relative change in VAD (to 15 mmHg, baseline) according to IOP elevation. The ocular perfusion pressure (OPP) was calculated as the difference between the MAP for each animal and IOP at each level. In all image analyses, two examiners (M.C. and S.W.K) carried out image analysis in a masked fashion.

### Statistical methods

SPSS (SPSS version 20.0 for Windows; IBM Corp., Armonk, NY, USA) was used for statistical analysis. Intraclass Correlation Coefficients (ICCs) were evaluated between two examiners for VAD measurement and the mean values of VAD from two examiners were used. Relative VAD in relation to increased IOP were compared with baseline (at 15 mmHg of IOP) using a repeated measure analysis of variance (ANOVA) test and a Wilcoxon signed rank test. P < 0.05 was considered to be significantly different.

## Supplementary Information

Supplementary Table 1.

## Data Availability

The raw data for this study is available upon reasonable request from the corresponding author.
