# Supplementary material for: Relationship between retinal capillary vessel density of OCT angiography and intraocular pressure in pig
Source: Sci Rep. 2021 Apr 20;11:8555. doi: 10.1038/s41598-021-87689-8 (PMC8058045; doi:10.1038/s41598-021-87689-8)
Supplement: Supplementary file 1 — Supplementary Table 1. [file 41598_2021_87689_MOESM1_ESM.docx]

**Relationship between retinal capillary vessel density of OCT angiography and intraocular pressure in pig**

Mihyun Choi^1^, Seong-Woo Kim^1,*^, Somin Ahn^1^, Vu Thi Que Anh^1,2^, Cheolmin Yun^3^, Yong Yeon Kim^1,**^

^1^Department of Ophthalmology, Korea University Guro Hospital, 148, Gurodong-ro, Guro-gu, Seoul 08308, Republic of Korea

^2^ Department of Ophthalmology, Hanoi Medical University, Hanoi, Vietnam

^3^Department of Ophthalmology, Korea University Ansan Hospital, 123, Jeokgeum-ro, Danwon-gu, Ansan-si, Gyeonggi-do, Republic of Korea

**Correspondence to**

*Professor Seong-Woo Kim, MD, PhD; ksw64723@korea.ac.kr

**Professor Yong Yeon Kim, MD, PhD: yongykim@korea.ac.kr

| **Supplementary table 1.** VAD before vitrectomy and baseline (15 mmHg) | | | | | |
| --- | --- | --- | --- | --- | --- |
|  | RBF | RPCs | SVP | IVP | DVP |
| Before vitrectomy | 0.441  (0.117) | 0.298 (0.097) | 0.290 (0.039) | 0.176 (0.060) | 0.112  (0.086) |
| Baseline  (15 mmHg) | 0.453  (0.092) | 0.285 (0.091) | 0.283 (0.043) | 0.152 (0.077) | 0.102 (0.054) |
| P-value* | 0.842 | 0.779 | 0.955 | 0.432 | 0.643 |
| All values are presented in mean (standard deviation).  VAD; Vessel area density  ^*^Wilcoxon singed-rank test, compared VAD between OCTA obtained prior to vitrectomy and baseline . | | | | | |
